# Antioxidant and Anti-Inflammatory Activity Determination of One Hundred Kinds of Pure Chemical Compounds Using Offline and Online Screening HPLC Assay

**DOI:** 10.1155/2015/165457

**Published:** 2015-10-04

**Authors:** Kwang Jin Lee, You Chang Oh, Won Kyung Cho, Jin Yeul Ma

**Affiliations:** Korean Institute of Oriental Medicine (KIOM), KM Application Center, 70 Cheomdan-ro, Dong-gu, Daegu 701-300, Republic of Korea

## Abstract

This study investigated the antioxidant activity of one hundred kinds of pure chemical compounds found within a number of natural substances and oriental medicinal herbs (OMH). Three different methods were used to evaluate the antioxidant activity of DPPH radical-scavenging activity, ABTS radical-scavenging activity, and online screening HPLC-ABTS assays. The results indicated that 17 compounds exhibited better inhibitory activity against ABTS radical than DPPH radical. The IC_50_ rate of a more practical substance is determined, and the ABTS assay IC_50_ values of gallic acid hydrate, (+)-catechin hydrate, caffeic acid, rutin hydrate, hyperoside, quercetin, and kaempferol compounds were 1.03 ± 0.25, 3.12 ± 0.51, 1.59 ± 0.06, 4.68 ± 1.24, 3.54 ± 0.39, 1.89 ± 0.33, and 3.70 ± 0.15 *μ*g/mL, respectively. The ABTS assay is more sensitive to identifying the antioxidant activity since it has faster reaction kinetics and a heightened response to antioxidants. In addition, there was a very small margin of error between the results of the offline-ABTS assay and those of the online screening HPLC-ABTS assay. We also evaluated the effects of 17 compounds on the NO secretion in LPS-stimulated RAW 264.7 cells and also investigated the cytotoxicity of 17 compounds using a cell counting kit (CCK) in order to determine the optimal concentration that would provide an effective anti-inflammatory action with minimum toxicity. These results will be compiled into a database, and this method can be a powerful preselection tool for compounds intended to be studied for their potential bioactivity and antioxidant activity related to their radical-scavenging capacity.

## 1. Introduction

Natural substances and oriental medicinal herbs (OMH) have been traditionally administered to treat or prevent various diseases in Asia, including Korea, China, and Japan [1]. Generally OMH have very effective anticancer, anti-inflammatory, and antivirus properties [2], and researchers have reported that these natural substances also exhibit antioxidant activity. In addition, their long historical clinical practice and reliable therapeutic efficacy make them excellent sources to discover natural bioactive compounds [3]. OMH have received extensive attention for their use as drugs, functional foods, and cosmetic materials [1, 4]. An extraction solvent composed of water and ethanol is commonly used to extract the bioactive compounds from OMH with subsequent boiling and distillation to obtain useful components [5]. The chemical constituents of OMH have been shown to be composed of natural products, including triterpenes, steroids, alkaloids, flavonoids, and polysaccharides [3, 6]. During our investigation on the potential antioxidant activity of the commonly known phytochemical, one hundred kinds of pure compounds were identified. Reactive oxygen species (ROS), which originate from oxygen, are naturally produced by some enzymes as part of the metabolism within the cytoplasm [7, 8]. However, excess ROS have a fatal effect on oxygen toxicity and cellular dysfunction. In addition, excess ROS have also been linked to maladies such as cancer, stroke, Parkinson's disease, heart disease, arteriosclerosis, infection, ageing, and autoimmune disease [8, 9]. Many studies have been carried out on the antioxidant activity that eliminates ROS to obtain more conclusive information [10, 11], and OMH have been reported to contain these kinds of antioxidants: ABTS, DPPH, and lipid peroxidation inhibition. The corresponding target compounds were used to identify the antioxidant activity, especially using DPPH or ABTS radical technique [12]. Recently, sensitive online HPLC methods (online HPLC-DPPH and online HPLC-ABTS assays) have been developed to analyse free radical-scavenging activity [5, 13]. An online system has been introduced to rapidly determine the antioxidant activity of each component in the given compounds, and online screening HPLC postcolumn assay involving DPPH or ABTS radical techniques has been developed to provide a new analysis screening technology method with which the bioactive compounds can be spectrophotometrically monitored due to the decrease in absorbance at 515 or 734 nm [14]. This new method was successfully applied to screen and identify the natural bioactivity of complex mixtures, especially for OMH [15]. In this study, we conduct DPPH and ABTS assays to screen for the antioxidant activity of one hundred kinds of pure chemical compounds, so the IC_50_ rate of a more practical substance is determined. We also evaluated the cytotoxicity of 17 compounds, including (1) (+)-catechin hydrate, (2) calycosin, (3) caffeic acid, (4) curcumin, (5) eugenol, (6) ferulic acid, (7) gallic acid hydrate, (8) hyperoside, (9) kaempferol, (10) magnolol, (11) quercetin, (12) quercetin 3-beta-D-glucoside, (13) quercitrin hydrate, (14) rutin hydrate, (15) sinapic acid, (16) vanillylacetone, and (17) L-(+)-ascorbic acid, by using a CCK assay to determine the optimal concentration that would be effective for anti-inflammatory activity with a minimum toxicity [9, 16]. In addition, the results of an online HPLC-ABTS assay of some of the compounds were compared and analysed to find a more practical approach toward the use of online screening HPLC-ABTS assays to quickly pinpoint peaks in chromatograms that correspond to bioactive compounds.

## 2. Experimental

### 2.1. Reagents and Materials

**Figure 1:**
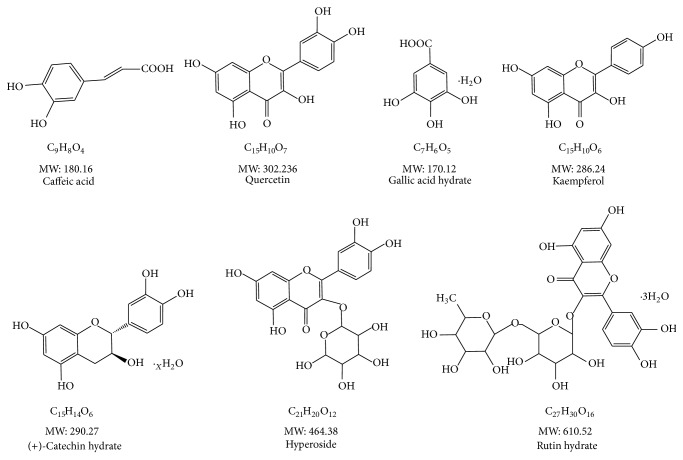
Chemical structure of the superior antioxidant activity compounds.

One hundred kinds of pure chemical compounds were purchased from KFDA (Korea), Daejung (Korea), Sigma (USA), Chem Faces (China), TCI (Japan), ChromaDex (USA), Fluka (USA), Wako (Japan), GlycoSyn (New Zealand, USA), Santa Cruz Biotech (USA), China Lang Chem Inc. (China), and RD Chemical (USA). The following reagents were used for radical-scavenging assays: ABTS (2,2′-azino-bis(3-ethylbenzothiazoline-6-sulfonic acid)), DPPH (2,2-diphenyl-1-picrylhydrazyl), potassium persulfate, and trifluoroacetic acid (TFA) were purchased from Sigma (USA). The HPLC-grade methanol and acetonitrile were purchased from J. T. Baker (USA). The triple distilled water was filtered with a 0.2 *μ*m membrane filter (Advantec, Tokyo, Japan) before analysis. Materials for cell culture were obtained from Lonza (Basel, Switzerland). LPS, bovine serum albumin (BSA), and 3-(4,5-dimethylthiazol-2-yl)-2,5-diphenyltetrazolium bromide (MTT) were purchased from Sigma (St. Louis, MO, USA). Antibodies for ELISA were obtained from eBioscience (San Diego, CA, USA). The chemical structures of potentially selected compounds are shown in Figure 1.

### 2.2. Standard Sample Preparation

The high purity standard sample (higher than >95%) was prepared by dissolving 2 mg of each standard chemical in 20 mL of methanol and adjusting the concentration to 100 *μ*g/mL.

### 2.3. Offline DPPH Assay for Antioxidant Activity Evaluation

The DPPH radical cation method [17] was modified to evaluate the free radical-scavenging effect of one hundred pure chemical compounds. The DPPH reagent was DPPH (8 mg) dissolved in MeOH (100 mL) for a solution concentration of 80 *μ*L/mL. To determine the scavenging activity, 100 *μ*L DPPH reagent was mixed with 100 *μ*L of sample in a 96-well microplate and was incubated at room temperature for 30 min. After incubation, the absorbance was measured 514 nm using an ELISA reader (TECAN, Gröding, Austria), and 100% methanol was used as a control. The DPPH scavenging effect was measured using the following formula:

(1) Radical  scavenging%=Acontrol−AsampleAcontrol×100.

The IC_50_ DPPH values (the concentration of sample required for inhibition of 50% of DPPH radicals) were obtained through extrapolation from regression analysis. The antioxidant was evaluated based on this IC_50_ value.

### 2.4. Offline-ABTS Assay for Antioxidant Activity Evaluation

The ABTS radical cation method [17] was modified to evaluate the free radical-scavenging effect of one hundred pure chemical compounds. The ABTS reagent was prepared by mixing 5 mL of 7 mM ABTS with 88 *μ*L of 140 mM potassium persulfate. The mixture was then kept in the dark at room temperature for 16 h to allow free radical generation and was then diluted with water (1 : 44, v/v). To determine the scavenging activity, 100 *μ*L ABTS reagent was mixed with 100 *μ*L of sample in a 96-well microplate and was incubated at room temperature for 6 min. After incubation, the absorbance was measured 734 nm using an ELISA reader (TECAN, Gröding, Austria), and 100% methanol was used as a control. The ABTS scavenging effect was measured using the following formula:

(2) Radical  scavenging%=Acontrol−AsampleAcontrol×100.

The IC_50_ ABTS values (the concentration of sample required for inhibition of 50% of ABTS radicals) were obtained through extrapolation from regression analysis. The antioxidant activity was evaluated based on this IC_50_ value.

### 2.5. Online Screening HPLC-ABTS Analysis

**Figure 2:**
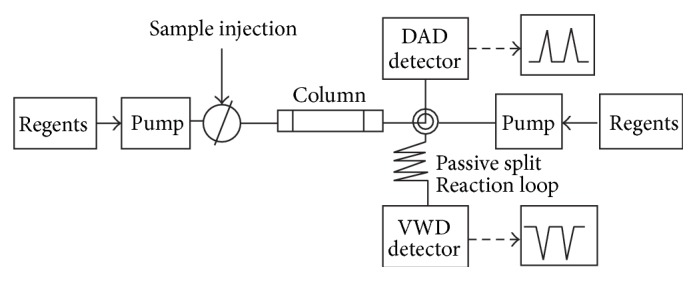
Schematic of online screening HPLC-ABTS system.

The online radical-scavenging activity of one hundred kinds of pure standard compounds was determined using the ABTS assay modifying the methods used by Stewart et al. [18]. A 2 mM ABTS stock solution containing 3.5 mM potassium persulfate was prepared and was kept in the dark at room temperature for 16 h to allow the completion of radical generation and was then diluted with water (1 : 29, v/v). Each pure sample was injected into a Dionex Ultimate 3000 HPLC system (Thromo Scientific). The chromatographic columns used in this experiment are commercially available; this is obtained from RS-tech (0.46 × 25 cm, 5 *μ*m, C_18_, Daejeon, Korea). The injection volume was 10 *μ*L, and the flow rate of the mobile phase was 1.0 mL/min. The wavelength of the UV detector was fixed at 203, 254, and 320 nm. The composition of the mobile phases was as follows: A, water/trifluoroacetic acid = 99.9/0.1, vol%, and B, acetonitrile 100%. The run time was 70 min and the solvent program was the linear gradient method (90 : 10–60 : 40, A : B vol%).  Figure 2 is a schematic showing the online coupling of HPLC to a DAD (diode array detector) and the continuous flow ABTS assay. Online HPLC then arrived at a “T” piece, where ABTS was added. The ABTS flow rate was 0.5 mL/min, delivered by a Dionex Ultimate 3000 Pump. After mixing through a 1 mL loop which was maintained at 40°C, the absorbance was measured by a VIS detector at 734 nm. Data were analyzed using Chromeleon 7 software.

### 2.6. Cell Culture and Drug Treatment

RAW 264.7 cells were obtained from the American Type Culture Collection (ATCC, Manassas, VA, USA) and grown in RPMI 1640 medium containing 10% FBS and 100 U/mL of antibiotics sulfate. The cells were incubated in humidified 5% CO_2_ atmosphere at 37°C. To stimulate the cells, the medium was changed with fresh RPMI 1640 medium and LPS (200 ng/mL) [18, 19] was added in the presence or absence of 17 compounds (1, 5, and 10 *μ*g/mL) for 24 h.

### 2.7. Cell Viability Assay

Cytotoxicity was analyzed using CCK (Dojindo, Japan). 17 compounds were added to the cells and incubated for 24 hours at 37°C with 5% CO_2_. 10 *μ*L CCK solutions were added to each well and the cells were incubated for another 1 h. Then the optical density was read at 450 nm using an ELISA reader (Infinite M200, Tecan, Männedorf, Switzerland).

### 2.8. Measurement of NO Production

NO production was analyzed by measuring the nitrite in the supernatants of cultured macrophage cells. The cells were pretreated with 17 compounds and stimulated with LPS for 24 hours. The supernatant was mixed with the same volume of Griess reagent (1% sulfanilamide, 0.1% naphthylethylenediamine dihydrochloride, and 2.5% phosphoric acid) and incubated at room temperature (RT) for 5 min [19]. The absorbance at 570 nm was read.

### 2.9. Inflammatory Cytokine Determination

Cells were seeded at a density of 5 × 10^5^ cells/mL in 24-well culture plates and pretreated with three concentrations of 17 compounds for 1 hour before LPS stimulation. ELISA plates (Nunc, Roskilde, Denmark) were coated overnight at 4°C with capture antibody diluted in coating buffer (0.1 M carbonate, pH 9.5) and then washed five times with phosphate-buffered saline (PBS) containing 0.05% Tween 20. The nonspecific protein-binding sites were blocked with assay diluent buffer (PBS containing 10% FBS, pH 7.0) for more than 1 hour. Promptly, samples and standards were added to the wells. After overnight of incubation at 4°C, the working detector solution (biotinylated detection antibody and streptavidin-HRP reagent) was added and incubated for 1 hour. Subsequently, substrate solution (tetramethylbenzidine) was added to the wells and incubated for 30 min in darkness before the reaction was stopped with stop solution (2 N H_3_PO_4_). The optical density was read at 450 nm [19].

### 2.10. Statistical Analysis

The results are expressed as mean ± SD values for the number (*n* = 3 times) of experiments. Statistical significance was compared for each treated group with the control and determined by Student's *t*-tests. Each experiment was repeated at least three times to yield comparable results. Values with *p* < 0.01 and *p* < 0.001 were considered significant.

## 3. Result and Discussion

Several researches have revealed that a variety of natural and chemical compounds from natural substance crops, fruits, vegetables, and oriental medicinal herbs (OMH) have shown high antioxidant activity after the extraction and purification processes [3]. In addition, various methods have been used to determine the antioxidant activity of natural substance crops, foods, and plant products [1, 4]. The present study used three different methods to evaluate the antioxidant activity consisting of DPPH radical-scavenging activity, ABTS radical-scavenging activity, and online screening HPLC-ABTS assays. Therefore, this work documented for the first time a comparison of the antioxidant activities of one hundred kinds of pure chemical compounds.

### 3.1. Offline DPPH and ABTS Assay

Antioxidant activity reportedly has an effect on various different bioactivities (whitening, anti-inflammation, and high blood pressure). The antioxidant activity of natural substances and OMH has been widely studied, and, thus, this study identifies the antioxidant activity of standard substances that have originated from various OMH in terms of their DPPH radical-scavenging activity and ABTS radical-scavenging activity. The DPPH and ABTS radical-scavenging assays offer a redox-functioned proton ion for unstable free radicals and play a critical role in stabilizing detrimental free radicals in the human body. This is generally achieved by taking advantage of the fact that unstable violet DPPH and ABTS free radicals transform to stable yellow DPPH free radicals by accepting a hydrogen ion from antioxidants. In terms of the antioxidant activity, the ability to eliminate hydroxyl radicals or superoxide radicals through a physiologic action or through oxidation is evaluated, and a high index indicates a strong antioxidant activity.  Table 1 provides the results of the DPPH and ABTS radical scavenging in 100 ppm for one hundred kinds of pure standard compounds used in this study. 17 compounds among the one hundred kinds of pure standard compounds ((1) (+)-catechin hydrate, (2) calycosin, (3) caffeic acid, (4) curcumin, (5) eugenol, (6) ferulic acid, (7) gallic acid hydrate, (8) hyperoside, (9) kaempferol, (10) magnolol, (11) quercetin, (12) quercetin 3-beta-D-glucoside, (13) quercitrin, (14) rutin hydrate, (15) sinapic acid, (16) vanillylacetone, and (17) L-(+)-ascorbic acid) have an antioxidant activity of over 90%.  Table 2 shows the IC_50_ rate of compounds with a strong antioxidant activity. The ABTS radical-scavenging measurement method, which is commonly used to evaluate the antioxidant activity, takes advantage of the fact that ABTS free radicals become stable by accepting a hydrogen ion from the antioxidant, losing their blue colors. Moreover, in the ABTS assay as well as in the DPPH assay, when antioxidant activity occurs, the ability to eliminate hydroxyl radicals or superoxide radicals through physiologic action or oxidation is evaluated with a high index indicating a strong antioxidant activity. Each of the DPPH and ABTS are compounds that have a proton free radical, with a characteristic absorption that decreases significantly upon exposure to proton radical scavengers. DPPH and ABTS radical-scavenging through antioxidant activity are well known to be attributable to their hydrogen-donating ability (Tables 1 and 2). The concentration of these compounds required to inhibit 50% of the radical-scavenging effect (IC_50_) has been determined by testing a series of concentrations. In particular, the sample with (+)-catechin hydrate, caffeic acid, eugenol, gallic acid hydrate, hyperoside, quercetin, vanillylacetone, and L-(+)-ascorbic acid compounds showed the strongest activity. In addition, the 17 compounds showed better inhibitory activity against ABTS radical than the DPPH radicals. That is, the ABTS assay is more sensitive in identifying antioxidant activity because of the faster reaction kinetics, and its response to antioxidants is higher. Consequently, this study shows that the ABTS assay IC_50_ values of gallic acid hydrate, (+)-catechin hydrate, caffeic acid, rutin hydrate, hyperoside, quercetin, and kaempferol compounds were 1.03 ± 0.25, 3.12 ± 0.51, 1.59 ± 0.06, 4.68 ± 1.24, 3.54 ± 0.39, 1.89 ± 0.33, and 3.70 ± 0.15 *μ*g/mL, respectively.

### 3.2. Online HPLC-ABTS Assay Analysis

**Figure 3:**
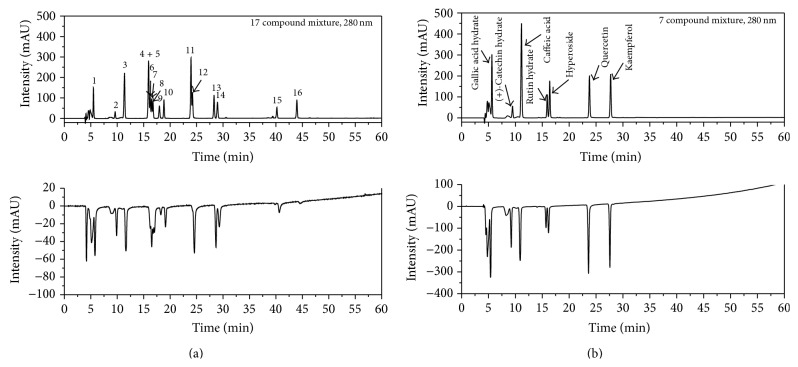
Identification antioxidant activity of online screening HPLC-ABTS assay ((a) simultaneous analysis of 17 compounds, (1) gallic acid hydrate, (2) (+)-catechin hydrate, (3) caffeic acid, (4) ferulic acid, (5) rutin hydrate, (6) sinapic acid, (7) hyperoside, (8) quercetin 3-*β*-D-glucoside, (9) vanillylacetone, (10) quercitrin hydrate, (11) calycosin, (12) quercetin, (13) kaempferol, (14) eugenol, (15) curcumin, (16) magnolol, and (17) ascorbic acid (not detected); (b) simultaneous analysis of 7 compounds).

The most popular approach utilises a relatively stable, coloured radical solution of DPPH or ABTS, which is added postcolumn to the HPLC flow. Drug, food, functional material, and plant and OMH samples are evaluated for their antioxidant capacities according to a variety of antioxidant activity test methods, such as those for ABTS [2,2′-azino-bis(3-ethylbenzothiazoline-6-sulfonic acid)] radical scavenging [20]. The HPLC analyses react postcolumn with the ABTS, and the reduction is detected as a negative peak by a VIS absorbance detector at 734 nm. The ABTS radical is much more water soluble than DPPH [13], so ABTS better shows the details of an online HPLC-ABTS assay system that analysed the 17 given compounds (Figures 3(a) and 3(b)). Combined UV (positive signals) and ABTS quenching (negative signals) chromatograms of the 17 compounds ((1) gallic acid hydrate *R*_*t*_: 5.46 min, (2) (+)-catechin hydrate *R*_*t*_: 9.26 min, (3) caffeic acid *R*_*t*_: 11.12 min, (4) ferulic acid *R*_*t*_: 15.87 min, (5) rutin hydrate *R*_*t*_: 15.99 min, (6) sinapic acid *R*_*t*_: 16.15 min, (7) hyperoside *R*_*t*_: 16.49 min, (8) quercetin 3-beta-D-glucoside *R*_*t*_: 16.82 min, (9) vanillylacetone *R*_*t*_: 17.92 min, (10) quercitrin *R*_*t*_: 18.90 min, (11) calycosin *R*_*t*_: 23.81 min, (12) quercetin *R*_*t*_: 24.31 min, (13) kaempferol *R*_*t*_: 28.37 min, (14) eugenol *R*_*t*_: 28.98 min, (15) curcumin *R*_*t*_: 40.20 min, (16) magnolol *R*_*t*_: 43.98 min, and (17) L-(+)-ascorbic acid) (not detected; L-(+)-ascorbic acid) (each concentration 100 ppm) are presented in Figure 3(a). Of these, seven compounds that showed excellent activity were further analysed. Several eluted substances were detected in the 7 compounds, including (1) gallic acid hydrate (210 nm), (2) (+)-catechin hydrate (210 nm), (3) caffeic acid (320 nm), (4) rutin hydrate (210 nm), (5) hyperoside (210 nm), (6) quercetin (210 nm), and (7) kaempferol (254 nm), which are observed as a positive signal on the UV detector (210, 254, and 320 nm). The retention times (*R*_*t*_) of (1) gallic acid hydrate (*R*_*t*_ 5.62 min), (2) (+)-catechin hydrate (*R*_*t*_ 9.46 min), (3) caffeic acid (*R*_*t*_ 11.12 min), (4) rutin hydrate (*R*_*t*_ 15.86 min), (5) hyperoside (*R*_*t*_ 16.26 min), (6) quercetin (*R*_*t*_ 23.58 min), and (7) kaempferol (*R*_*t*_ 28.30 min) are reported in Figure 3(b). The other compounds exhibited a hydrogen-donating capacity (negative peak) towards the ABTS radical at the applied concentration. These results therefore reveal that this method can be applied for quick screening of antioxidant activity or, more precisely, of radical-scavenging activity (Table 3). This work confirms the feasibility of assessing the bioactivity of specific phytochemicals by using an online screening HPLC-ABTS assay. This method was successfully applied to screen and identify the antioxidant activity of natural substances and OMH complex mixtures [5, 15]. The results show the shape of the chromatogram by the competitive adsorption and desorption. In addition, the screening methods for the rapid activity can provide useful information in basic research on natural products chemistry and isolation analysis. It is considered that the data will only be valuable in engineering and also very useful as functional materials and pharmaceutical materials in commercial processes.

### 3.3. Anti-Inflammatory Activity Screening

#### 3.3.1. Effect of 17 Compounds on RAW 264.7 Cell Viability

**Figure 4:**
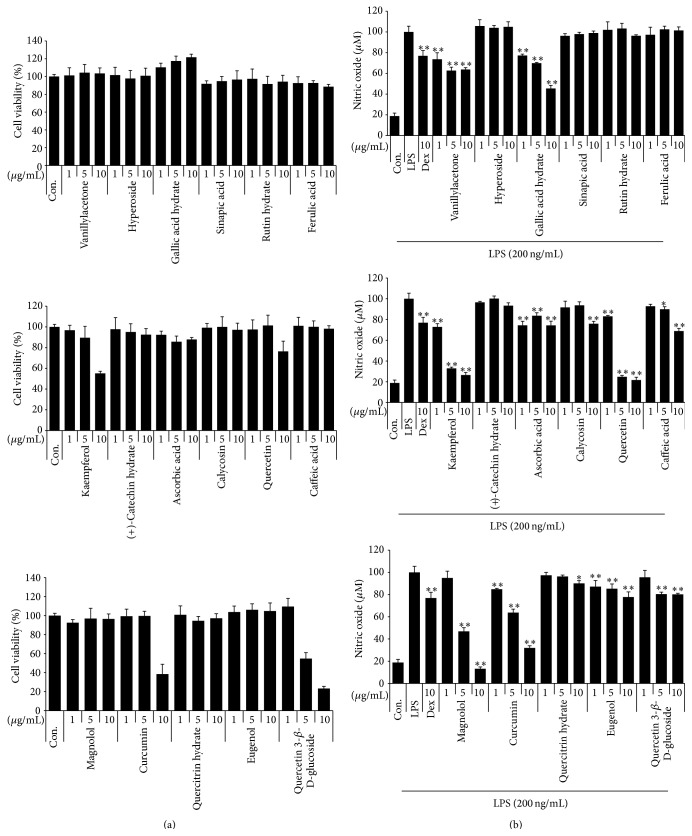
Effect of 17 compounds on (a) cell viability and LPS-induced (b) NO production in RAW 264.7 cells. RAW 264.7 cells were pretreated with 17 compounds for 1 hour before incubation with LPS for 24 hours. (a) Cytotoxicity was evaluated by a CCK. (b) The culture supernatant was analyzed for nitrite production. As a control, the cells were incubated with vehicle alone. Data shows mean ± SE values of triplicate determination from independent experiments. ^*∗*^ *p* < 0.01 and ^*∗∗*^ *p* < 0.001 were calculated from comparing with LPS-stimulation value.

We evaluated the cytotoxicity of the 17 compounds by using CCK to determine the optimal concentration that would be effective in providing anti-inflammatory activity with a minimum toxicity. As shown in Figure 4(a), kaempferol, quercetin, and curcumin show toxicity at a concentration of 10 *μ*g/mL. Also, quercetin 3-beta-D-glucoside has a strong toxicity on macrophage viability at 5 *μ*g/mL or more. Vanillylacetone, hyperoside, gallic acid hydrate, sinapic acid, rutin hydrate, ferulic acid, (+)-catechin hydrate, ascorbic acid, calycosin, caffeic acid, magnolol, quercitrin hydrate, and eugenol did not affect cell viability up to 10 *μ*g/mL, indicating that these 13 compounds are not toxic to cells.

#### 3.3.2. Effect of the 17 Compounds on NO Production in LPS-Stimulated RAW 264.7 Macrophages

We evaluated the effects of 17 compounds on NO secretion in LPS-stimulated RAW 264.7 cells. The cells were pretreated with 17 compounds at concentrations of 1, 5, and 10 *μ*g/mL prior to LPS stimulation, and NO production was also measured. We employed 10 *μ*M dexamethasone as a positive control, since it is widely used as an anti-inflammatory agent. As shown in Figure 4(b), vanillylacetone, gallic acid hydrate, kaempferol, quercetin, magnolol, and curcumin exhibit a strong inhibitory effect on NO secretion upon LPS stimulation. The inhibitory effects of 10 *μ*g/mL kaempferol, quercetin, and curcumin on NO production were a result of their cytotoxicity. However, kaempferol, quercetin, and curcumin exert effective inhibition at concentrations of 1 and 5 *μ*g/mL. In particular, magnolol strongly inhibited NO production in a dose-dependent manner without toxicity. Hyperoside, sinapic acid, rutin hydrate, ferulic acid, (+)-catechin hydrate, ascorbic acid, calycosin, caffeic acid, quercitrin hydrate, eugenol, and quercetin 3-beta-D-glucoside do not show remarkable suppressive effects.

#### 3.3.3. Effect of the 17 Compounds on LPS-Induced Inflammatory Cytokines Production

**Figure 5:**
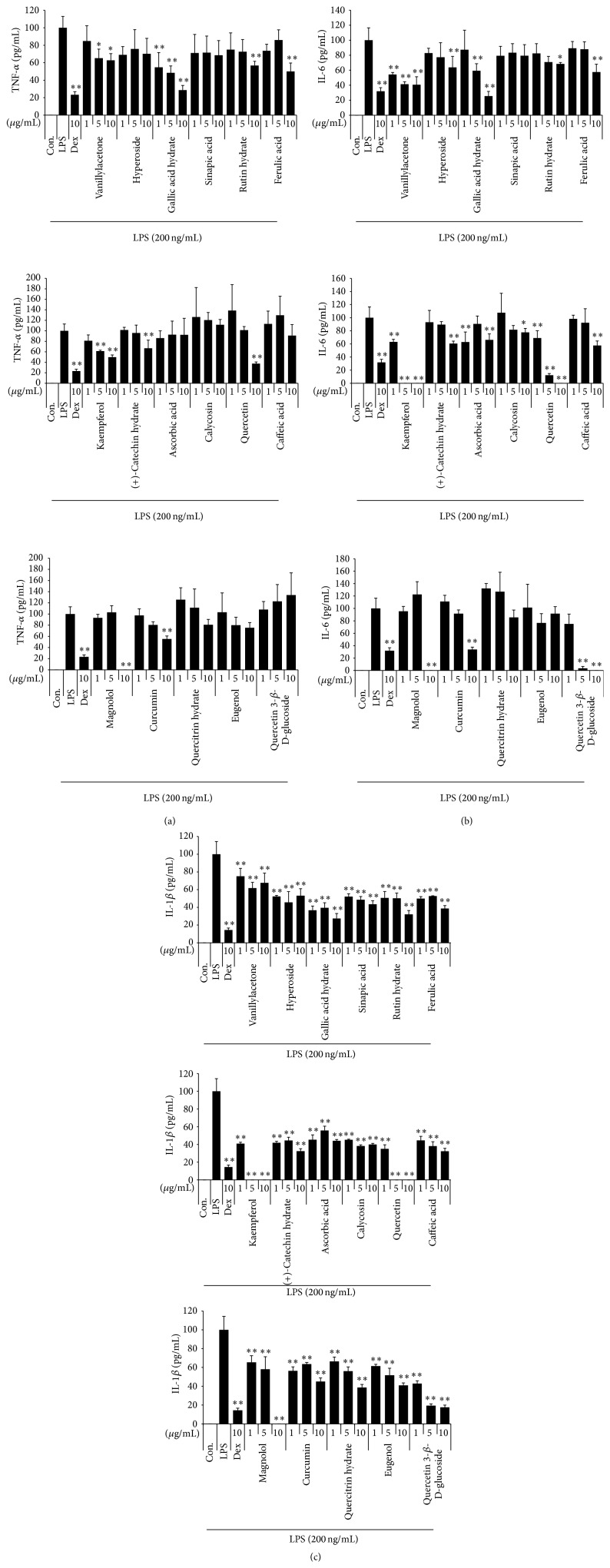
Effect of 17 compounds on the production of (a) TNF-*α*, (b) IL-6, and (c) IL-1*β* cytokine in macrophages. Cells were pretreated with 17 compounds for 1 hour before being incubated with LPS for 24 hours. Production of cytokines was measured by ELISA. Data shows mean ± SE values of triplicate determinations from three independent experiments. ^*∗*^ *p* < 0.01 and ^*∗∗*^ *p* < 0.001 were calculated from comparing with LPS-stimulation value.

Next, we investigated the inhibitory effect of the 17 compounds on the production of inflammatory cytokines, including TNF-*α*, IL-6, and IL-1*β*, which are the other parameters of the inflammation. Gallic acid hydrate exerts an inhibitory effect on the TNF-*α* cytokine production at all concentrations in a dose-dependent manner. In addition, 5 *μ*g/mL kaempferol and 10 *μ*g/mL magnolol show a strong inhibitory effect (Figure 5(a)). As shown in Figure 5(b), vanillylacetone, gallic acid hydrate, kaempferol, and quercetin significantly inhibited IL-6 cytokine secretion in a statistically significant, dose-dependent manner. In addition, all of the compounds showed an inhibitory effect on IL-1*β* cytokine production. Gallic acid hydrate, kaempferol, quercetin, and magnolol were especially strong inhibitors of IL-1*β* cytokine production in a dose-dependent manner (Figure 5(c)).

## 4. Conclusions

This study provides a comparison of the free radical scavengers in the one hundred kinds of pure chemical compounds through an offline DPPH radical-scavenging activity assay, ABTS radical-scavenging activity assay, and an online screening HPLC-ABTS assay. Here, the IC_50_ rate of a more practical substance is determined. The results indicate that the ABTS assay IC_50_ values of gallic acid hydrate, (+)-catechin hydrate, caffeic acid, rutin hydrate, hyperoside, quercetin, and kaempferol compounds were 1.03 ± 0.25, 3.12 ± 0.51, 1.59 ± 0.06, 4.68 ± 1.24, 3.54 ± 0.39, 1.89 ± 0.33, and 3.70 ± 0.15 *μ*g/mL, respectively. This testing methodology provided a useful tool to focus efforts on chemically active radical-scavenging compounds with high kinetic rates and allowed quick gathering of useful information related to the molecular compounds in terms of their antioxidant activity potential. In addition, there was a very small margin of error between the results of the offline-ABTS assay and those of the online screening HPLC-ABTS assay. We also evaluated the effects of 17 compounds on NO secretion in LPS-stimulated RAW 264.7 cells and the cytotoxicity of the 17 compounds using CCK to determine the optimal concentration that would be effective to provide anti-inflammatory activity with a minimum toxicity. These results will be compiled into a database, and this method can therefore be a powerful preselection tool for compounds intended to be studied for their potential bioactivity and antioxidant activity related to their radical-scavenging capacity.

## Figures and Tables

**Table 1:** Free radical-scavenging capacities of antioxidant activity available measured with DPPH and ABTS assay on microwell plate.

Number	Compounds names	Compounds purchased	Concentration *μ*M (*μ*mol/L)	Radical scavenging (%)	
				DPPH	ABTS
1	Albiflorin	Wako	208.13	−0.15 ± 0.39	0.14 ± 5.27
2	Alisol A	Wako	203.79	−0.47 ± 0.71	12.92 ± 0.86
3	Alisol B	Wako	211.55	−1.66 ± 0.23	12.78 ± 1.35
4	Amygdalin	KFDA	218.61	−0.85 ± 0.67	12.31 ± 0.03
5	Anthraquinone	Wako	480.28	0.12 ± 0.70	0.86 ± 5.12
6	Atractylenolide iii	Chem Faces	402.71	−2.07 ± 0.53	1.34 ± 8.29
7	Aucubin	Wako	288.74	−1.64 ± 0.79	1.22 ± 9.74
8	Baicalein	KFDA	370.04	95.84 ± 0.15	99.37 ± 0.12
9	Benzoic acid	Sigma	818.87	1.88 ± 0.42	3.80 ± 0.26
10	Berberine	Chem Faces	297.30	0.64 ± 0.43	84.68 ± 2.55
11	Berberine HCl	KFDA	268.95	−0.30 ± 0.30	8.74 ± 8.38
12	Caffeic acid	Sigma	555.06	95.91 ± 0.16	99.66 ± 0.24
13	Calycosin	Chem Faces	351.79	64.51 ± 0.59	99.19 ± 0.05
14	Catalpol	Wako	275.99	−2.55 ± 0.47	1.12 ± 9.62
15	Chrysin	Sigma	393.33	0.45 ± 0.53	99.13 ± 0.06
16	Cimifugin	Chem Faces	326.46	−0.89 ± 0.21	14.39 ± 1.81
17	Cinnamyl alcohol	Sigma	745.27	−0.03 ± 0.37	15.18 ± 0.75
18	cis-Inositol	Sigma	555.06	0.15 ± 0.26	0.54 ± 1.22
19	Costunolide	Sigma	430.44	2.86 ± 0.17	20.13 ± 8.74
20	Crocin	Sigma	102.36	24.43 ± 1.28	47.73 ± 0.67
21	Curcumin	Sigma	271.46	97.50 ± 0.63	99.97 ± 0.16
22	(+)-Catechin hydrate	TCI	344.51	94.50 ± 0.16	99.15 ± 0.06
23	1,8-Dihydroxy-3-methylanthraquinone	Sigma	393.33	−0.94 ± 0.54	2.09 ± 2.60
24	D-(−)-Lactic acid	Sigma	1110.12	2.51 ± 2.40	28.29 ± 0.74
25	D-(+)-Chiro-inositol	Sigma	555.06	0.49 ± 0.33	0.21 ± 0.45
26	Daidzein	Wako	393.33	0.52 ± 0.61	99.49 ± 0.49
27	Decursin	KFDA	304.54	−3.01 ± 0.91	0.97 ± 2.75
28	Decursinol	Chem Faces	406.07	−1.59 ± 1.03	15.12 ± 1.81
29	Dioscin	Sigma	115.07	−2.93 ± 1.30	0.43 ± 2.75
30	Diosgenin	Sigma	241.18	−1.38 ± 0.70	1.96 ± 9.24
31	D-Pinitol	Sigma	514.99	−2.27 ± 0.33	12.72 ± 1.85
32	6,7-Dimethylesculetin	RD Chemical	484.99	−2.74 ± 0.54	1.31 ± 0.21
33	(−)-Epicatechin	Sigma	344.51	94.51 ± 0.41	99.61 ± 0.16
34	(−)-Epigallocatechin gallate	Sigma	218.16	95.69 ± 0.14	99.51 ± 0.24
35	Eleutheroside B	Wako	268.55	−2.04 ± 1.02	1.97 ± 2.85
36	Emodin	TCI	370.04	2.20 ± 1.16	91.27 ± 1.39
37	Ephedrine-HCl	KFDA	495.81	−2.11 ± 1.52	0.00 ± 0.12
38	Ergosterol	Chem Faces	252.11	−1.65 ± 1.34	0.55 ± 9.46
39	Eugenol	Sigma	609.01	93.72 ± 0.12	99.91 ± 0.55
40	Evodiamine	KFDA	329.64	4.21 ± 1.55	40.76 ± 2.09
41	Ferulic acid	Sigma	514.99	95.12 ± 0.24	98.96 ± 0.27
42	Gallic acid hydrate	TCI	587.82	95.56 ± 0.03	101.30 ± 2.12
43	Geniposide	Chem Faces	257.49	−1.45 ± 0.90	1.22 ± 9.49
44	Genistein	TCI	370.04	−1.52 ± 0.30	98.50 ± 0.48
45	Genistin	Wako	231.28	2.23 ± 0.64	100.65 ± 0.03
46	Geraniol	Sigma	112.49	−2.43 ± 1.67	14.94 ± 0.95
47	Glabridin	Wako	308.29	42.20 ± 0.88	100.58 ± 0.04
48	Glimepiride	Sigma	203.82	−1.33 ± 0.63	7.84 ± 2.30
49	Glycyrrhetic acid	TCI	212.46	0.66 ± 0.65	10.23 ± 0.62
50	Glycyrrhizin	TCI	121.52	1.59 ± 1.96	12.36 ± 1.15
51	Gomisin A	KFDA	240.12	1.80 ± 0.28	1.74 ± 0.22
52	Gomisin N	KFDA	249.71	0.45 ± 0.51	3.80 ± 0.21
53	Hesperidin	KFDA	202.23	31.84 ± 0.37	100.21 ± 0.01
54	Hyperoside	Chem Faces	215.34	93.16 ± 0.25	99.62 ± 0.20
55	2′-Hydroxy-4′-methoxy-acetophenone	Sigma	601.76	−2.81 ± 0.28	99.73 ± 1.04
56	Icariin	TCI	147.78	3.82 ± 1.08	14.60 ± 0.12
57	Imperatorin	Chem Faces	369.99	−0.71 ± 2.31	0.74 ± 9.51
58	Isoimperatorin	Santa Cruz Biotech	369.99	0.27 ± 0.22	3.06 ± 2.17
59	Jujuboside A	Chem Faces	82.83	−1.63 ± 0.35	0.26 ± 9.74
60	Kaempferol	Chem Faces	349.36	95.02 ± 0.22	99.84 ± 0.41
61	Liquiritigenin	Chem Faces	390.24	12.26 ± 0.86	0.12 ± 0.68
62	Loganin	KFDA	256.16	2.62 ± 1.28	5.80 ± 0.62
63	Magnolol	KFDA	375.47	54.50 ± 0.12	77.74 ± 1.06
64	Mevinolin	Sigma	247.19	−0.26 ± 0.07	2.78 ± 0.69
65	Morroniside	China Lang Chem Inc.	246.08	7.11 ± 0.58	19.62 ± 1.83
66	Naringin	Sigma	172.26	3.05 ± 0.37	100.36 ± 0.05
67	Nodakenin	Chem Faces	244.86	0.68 ± 0.45	15.70 ± 1.92
68	Oleanolic acid	Wako	218.96	−0.37 ± 0.54	0.00 ± 0.12
69	Ononin	Sigma	232.34	2.23 ± 1.58	22.91 ± 1.89
70	Oxymatrine	Chem Faces	378.26	−1.63 ± 0.17	0.72 ± 8.85
71	Oxypeucedanin	Chem Faces	349.31	−0.11 ± 0.27	19.10 ± 2.38
72	Paeoniflorin	TCI	208.13	4.29 ± 1.43	22.03 ± 0.91
73	Paeonol	Sigma	601.79	0.99 ± 2.21	19.79 ± 2.35
74	Palmatine chloride hydrate	Sigma	257.82	2.57 ± 2.42	84.15 ± 2.43
75	Palmatine	Chem Faces	292.05	0.12 ± 0.23	68.05 ± 3.63
76	p-Coumaric acid	Sigma	609.16	8.89 ± 0.04	38.52 ± 0.84
77	Poncirin	KFDA	168.19	−1.44 ± 1.27	84.20 ± 3.66
78	Puerarin	Wako	240.17	9.89 ± 0.16	100.62 ± 0.06
79	Quercetin	Sigma	330.86	96.02 ± 0.08	100.18 ± 0.06
80	Quercetin 3-*β*-D-glucoside	Sigma	215.34	94.43 ± 0.02	99.94 ± 0.06
81	Quercitrin hydrate	Sigma	223.03	93.32 ± 0.04	99.12 ± 0.14
82	Rutaecarpine	KFDA	348.04	−1.41 ± 0.60	97.23 ± 0.63
83	Rutin hydrate	Sigma	163.79	93.57 ± 0.13	100.10 ± 0.02
84	Saikosaponin a	KFDA	128.04	−0.67 ± 0.79	16.17 ± 1.62
85	Salicylaldehyde	Sigma	818.87	2.07 ± 0.04	100.36 ± 0.05
86	Schisandrin	KFDA	231.21	−2.39 ± 0.89	10.29 ± 2.43
87	Sennoside A	KFDA	115.91	−3.09 ± 0.64	87.86 ± 3.21
88	Sequoyitol	GlycoSyn	514.99	−3.20 ± 1.37	10.28 ± 2.07
89	Sinapic acid	Fluka	445.99	94.84 ± 0.33	99.99 ± 0.23
90	Spinosin	Chem Faces	164.33	−0.96 ± 2.26	80.16 ± 2.20
91	Tetrandrine	Fluka	160.58	60.83 ± 2.11	100.28 ± 0.06
92	trans-Cinnamaldehyde	Sigma	756.66	2.83 ± 0.69	19.00 ± 1.37
93	trans-Cinnamic acid	Sigma	674.95	0.19 ± 0.15	0.02 ± 9.45
94	Uric acid	Sigma	594.85	40.37 ± 1.97	98.33 ± 0.31
95	Vanillylacetone	Sigma	514.85	93.78 ± 0.06	99.92 ± 0.24
96	Wogonin	KFDA	351.79	1.02 ± 0.51	100.57 ± 0.13
97	Wogonoside	Chem Faces	217.21	10.84 ± 0.10	98.62 ± 0.32
98	Ziyuglycoside I	Chem Faces	130.38	−1.71 ± 1.00	0.01 ± 9.85
99	Z-Ligustilide	Chem Faces	531.29	1.92 ± 0.76	68.69 ± 2.14
100	Ascorbic acid (Vitamin C)	Daejung	567.79	99.56 ± 0.89	99.89 ± 0.04

**Table 2:** Antioxidant activity of 17 compounds with offline DPPH and ABTS IC_50_ assay.

Number	Name	Radical- scavenging IC_50_	
		(*μ*g/mL)	
		DPPH	ABTS
1	(+)-Catechin hydrate	5.25 ± 0.31	3.12 ± 0.51
2	Calycosin	61.88 ± 1.19	33.21 ± 3.59
3	Caffeic acid	4.50 ± 0.30	1.59 ± 0.06
4	Curcumin	8.89 ± 0.24	4.99 ± 0.45
5	Eugenol	5.22 ± 0.25	3.22 ± 0.45
6	Ferulic acid	9.49 ± 0.21	1.99 ± 0.12
7	Gallic acid hydrate	1.56 ± 0.38	1.03 ± 0.25
8	Hyperoside	5.44 ± 0.36	3.54 ± 0.39
9	Kaempferol	7.78 ± 0.30	3.70 ± 0.15
10	Magnolol	85.57 ± 1.40	8.37 ± 0.56
11	Quercetin	2.66 ± 0.24	1.89 ± 0.33
12	Quercetin 3-*β*-D-glucoside	7.05 ± 0.59	3.59 ± 0.89
13	Quercitrin hydrate	7.55 ± 0.77	4.23 ± 0.84
14	Rutin hydrate	9.72 ± 1.06	4.68 ± 1.24
15	Sinapic acid	8.26 ± 0.41	5.36 ± 0.85
16	Vanillylacetone	5.69 ± 0.00	3.45 ± 0.05
17	Ascorbic acid (Vitamin C)	3.65 ± 0.23	2.65 ± 0.46

**Table 3:** Simultaneous identification of antioxidant activity with online screening HPLC-ABTS assay.

Compounds	UV wavelength (nm)	Retention time (min)	Peak area (mAu)		Positive S.D.	Negative S.D.
			Positive (average)	Negative (average)		
Gallic acid hydrate	210	5.623	72.116	51.624	0.054	0.405
(+)-Catechin hydrate	210	9.463	75.974	57.981	0.076	0.328
Caffeic acid	320	11.123	108.475	57.808	0.048	0.433
Rutin hydrate	210	15.860	51.185	13.241	0.393	0.023
Hyperoside	210	16.263	80.346	15.631	0.017	0.213
Quercetin	210	23.583	109.672	22.155	0.101	0.067
Kaempferol	254	28.303	56.806	30.651	0.143	0.071

## References

[1] Ishimaru K., Nishikawa K., Omoto T., Asai I., Yoshihira K., Shimomura K. (1995). Two flavone 2′-glucosides from *Scutellaria baicalensis*. *Phytochemistry*.

[2] Park H. G., Yoon S. Y., Choi J. Y. (2007). Anticonvulsant effect of wogonin isolated from *Scutellaria baicalensis*. *European Journal of Pharmacology*.

[3] Lee J. H., Lee B. W., Kim B. (2013). Changes in phenolic compounds (Isoflavones and Phenolic acids) and antioxidant properties in high-protein soybean (*Glycine max* L., cv. Saedanbaek) for different roasting conditions. *Journal of the Korean Society for Applied Biological Chemistry*.

[4] Song X.-Y., Li Y.-D., Shi Y.-P., Jin L., Chen J. (2013). Quality control of traditional Chinese medicines: a review. *Chinese Journal of Natural Medicines*.

[5] Lee K. J., Choi S. D., Ma J. Y. (2013). Phytochemical analysis of curcumin from turmeric by RP-HPLC. *Asian Journal of Chemistry*.

[6] Hu L.-L., Ma Q.-Y., Huang S.-Z. (2013). Two new phenolic compounds from the fruiting bodies of *Ganoderma tropicum*. *Bulletin of the Korean Chemical Society*.

[7] Hazra B., Sarma M. D., Sanyal U. (2004). Separation methods of quinonoid constituents of plants used in Oriental traditional medicines. *Journal of Chromatography B*.

[8] Adegoke O., Forbes P. B. C. (2015). Challenges and advances in quantum dot fluorescent probes to detect reactive oxygen and nitrogen species: a review. *Analytica Chimica Acta*.

[9] Melo P. S., Massarioli A. P., Denny C. (2015). Winery by-products: extraction optimization, phenolic composition and cytotoxic evaluation to act as a new source of scavenging of reactive oxygen species. *Food Chemistry*.

[10] Kim K. S., Lee S. H., Lee Y. S. (2003). Anti-oxidant activities of the extracts from the herbs of *Artemisia apiacea*. *Journal of Ethnopharmacology*.

[11] Gao Z., Huang K., Yang X., Xu H. (1999). Free radical scavenging and antioxidant activities of flavonoids extracted from the radix of *Scutellaria baicalensis* Georgi. *Biochimica et Biophysica Acta—General Subjects*.

[12] Zhang Y., Li Q., Xing H. (2013). Evaluation of antioxidant activity of ten compounds in different tea samples by means of an on-line HPLC-DPPH assay. *Food Research International*.

[13] He W., Liu X., Xu H., Gong Y., Yuan F., Gao Y. (2010). On-line HPLC-ABTS screening and HPLC-DAD-MS/MS identification of free radical scavengers in Gardenia (*Gardenia jasminoides* Ellis) fruit extracts. *Food Chemistry*.

[14] Shi S.-Y., Zhang Y.-P., Jiang X.-Y. (2009). Coupling HPLC to on-line, post-column (bio)chemical assays for high-resolution screening of bioactive compounds from complex mixtures. *Trends in Analytical Chemistry*.

[15] Lee K. J., Song N.-Y., Oh Y. C., Cho W.-K., Ma J. Y. (2014). Isolation and bioactivity analysis of ethyl acetate extract from *Acer* tegmentosum using in vitro assay and on-line screening HPLC-ABTS^+^ system. *Journal of Analytical Methods in Chemistry*.

[16] Yu T., Lee J., Lee Y. G. (2010). *In vitro* and *in vivo* anti-inflammatory effects of ethanol extract from *Acer tegmentosum*. *Journal of Ethnopharmacology*.

[17] Pellegrini N., Ying M., Rice-Evans C. (1999). Screening of dietary carotenoids and carotenoid-rich fruits extract for antioxidant activities applying 2,2′-azobis (3-ethylbenzothine-6-surfonic acid) radical cation decolorization assay. *Methods in Enzymology*.

[18] Stewart A. J., Mullen W., Crozier A. (2005). On-line high-performance liquid chromatography analysis of the antioxidant activity of phenolic compounds in green and black tea. *Molecular Nutrition & Food Research*.

[19] Choi H.-J., Kang O.-H., Park P.-S. (2007). Mume Fructus water extract inhibits pro-inflammatory mediators in lipopolysaccharide-stimulated macrophages. *Journal of Medicinal Food*.

[20] Karaçelik A. A., Küçük M., Iskefiyeli Z. (2015). Antioxidant components of *Viburnum opulus* L. determined by on-line HPLC-UV-ABTS radical scavenging and LC-UV-ESI-MS methods. *Food Chemistry*.

